# Research progress in the role and mechanism of Leucine in regulating animal growth and development

**DOI:** 10.3389/fphys.2023.1252089

**Published:** 2023-11-17

**Authors:** Shahab Ur Rehman, Rahmat Ali, Hao Zhang, Muhammad Hammad Zafar, Mengzhi Wang

**Affiliations:** Laboratory of Metabolic Manipulation of Herbivorous Animal Nutrition, College of Animal Science and Technology, Yangzhou University, Yangzhou, China

**Keywords:** Leucine, animals growth regulator, anabolic effects, mTOR1, therapeutic potential

## Abstract

Leucine, a branched-chain amino acid, is essential in regulating animal growth and development. Recent research has uncovered the mechanisms underlying Leucine’s anabolic effects on muscle and other tissues, including its ability to stimulate protein synthesis by activating the mTORC1 signaling pathway. The co-ingestion of carbohydrates and essential amino acids enhances Leucine’s anabolic effects. Moreover, Leucine has been shown to benefit lipid metabolism, and insulin sensitivity, making it a promising strategy for preventing and treating metabolic diseases, including type 2 diabetes and obesity. While emerging evidence indicates that epigenetic mechanisms may mediate Leucine’s effects on growth and development, more research is needed to elucidate its mechanisms of action fully. Specific studies have demonstrated that Leucine promotes muscle growth and metabolic health in animals and humans, making it a promising therapeutic agent. However, it is essential to note that Leucine supplementation may cause digestive issues or interact with certain medications, and More study is required to determine definitively optimal dosages. Therefore, it is important to understand how Leucine interacts with other nutrients, dietary factors, and lifestyle habits to maximize its benefits. Overall, Leucine’s importance in human nutrition is far-reaching, and its potential to prevent muscle loss and enhance athletic performance warrants further investigation.

## 1 Introduction

Leucine is animal and plant proteins’ most abundant branched-chain amino acid (BCAA). It is essential in protein synthesis and metabolic function and is sometimes used as a dietary supplement or enhances food flavor (Li et al., 2009). The first BCAAs leucine was discovered by a French scientist from cheese in 1819 named Prust (Wu, 2021). In 1820, Braconnot was the first to use Acid hydrolysis to isolate Leucine from the skeletal muscle and wool. Schulze synthesized Leucine from isovaleraldehyde in 1935 (Karkashadze et al., 2023). It optimized Leucine, an isoleucine isomer essential for optimal growth development. Leucine, isoleucine, and valine, branched-chain amino acids [BCAAs], are essential amino acids that are potent nutritional signaling molecules for regulating protein synthesis that is not synthesized in mammals (McCOY et al., 2009). Leucine has a rich quality of protein foods in BCAAs. The [BCAAs] and the supplement of Leucine wildly used in various clinical situations to maintain muscle mass during weight loss (Zhang et al., 2016). Leucine regulates growth and development through various mechanisms, including protein synthesis, energy metabolism, and immune function. Increasing dietary leucine intake may be an effective strategy for promoting growth and development and maintaining overall health (Zhang et al., 2017). Leucine is an aliphatic isobutyl side chain. That’s ways leucine is classified as hydrophobic white crystalline Amino acid. The molecular formula of Leucine is C6H13NO2. The leucine BCAAs have a 337c melting point, and 5 .8 is an isoelectric point (Gerling et al., 2014). The effect of Leucine in modifying mTOR signaling is not entirely known. Conversely, Leucine promotes protein translation by activating the mechanistic target of the rapamycin complex 1 (mTORC1) pathway. Leucine plays a significant role in stimulating protein synthesis via the mammalian target of rapamycin [mTOR] signaling pathway, fatty acid oxidation, and mitochondrial in both skeletal muscle and adipose tissue (Duan et al., 2016; Li et al., 2011), including placental cells and epithelial cells (Duan et al., 2015). Leucine stimulates mTORC1 activation by bringing the protein to the surface of lysosomes, where growth factors and other amino acids may stimulate it. Multiple downstream targets of mTORC1 are phosphorylated, which stimulates translation initiation and ribosome biogenesis (Liu and Sabatini, 2020). In addition to its role in protein synthesis, leucine also plays a role in the oxidation of fatty acids in skeletal muscle. Leucine stimulates fatty acid oxidation by activating the AMP-activated protein kinase (AMPK) pathway. AMP-activated protein kinase (AMPK) is a cellular energy sensor that controls metabolic rate and energy homeostasis. Leucine prevents lipid buildup in skeletal muscle and increases fatty acid oxidation by activating AMPK (Doi et al., 2005). The mitochondrial function is also affected by leucine. The oxidative phosphorylation process, which produces ATP, occurs in a cell’s mitochondria. Leucine promotes mitochondrial biogenesis, which boosts cellular mitochondrial content and energy production. Leucine promotes mitochondrial biogenesis and function by activating the peroxisome proliferator-activated receptor gamma coactivator 1-alpha (PGC-1) pathway. Notably, the mTOR pathway significantly impacts the development of mRNA translation and ribosome biogenesis, which need significant amounts of cellular energy (Daher et al., 2022). There is a lot of research on the possible health advantages of leucine supplementation, especially regarding muscle growth, metabolic function, and immunological function. But there are also hazards linked to supplementation, so it is vital to weigh the pros and cons before deciding whether or not to add leucine to your diet (Bauer et al., 2013). There is evidence that leucine may promote muscle protein synthesis and enhance muscle growth in healthy people and those with specific medical diseases like cancer or age-related muscle loss (Ham et al., 2014). One study showed that participants ranged in age from 65 to 82 years old, and the research was a randomized, double-anonymized, placebo-controlled trial of 16 healthy older individuals (8 women and 8 men). Supplementation with leucine or a placebo was given to participants for 6 weeks. Participants took the leucine supplement three times a day, with each dose containing 2.5 g of leucine (Walker et al., 2020). Individuals in the study who took leucine supplements gained significantly more muscle mass than those in the placebo group. Leucine supplementation increased total lean body mass and arm lean mass, but placebo had no effect. The leucine group also saw a significant boost in muscle strength compared to the placebo group, and supplemental leucine may help healthy older people gain muscle and strength (Yoshimura et al., 2019). Further studies are required to evaluate leucine supplementation’s ideal quantities, timing, and long-term effects. One investigation of the long-term effects of leucine supplementation in elderly individuals with sarcopenia. The study’s findings revealed that compared to the control group, the individuals who got the leucine supplement had significantly more muscular mass and strength (Bauer et al., 2015). Physical abilities, including gait speed and stair climbing capability, were improved in the leucine group. Leucine group members also saw a decline in indicators of inflammation, which are often linked to muscle wasting and weakness in older people. So, Research shows that healthy older adults who take supplemental leucine have gains in muscle growth and strength (Liberman et al., 2019). Research on the long-term benefits of leucine supplementation, especially for those with sarcopenia or other muscle-wasting diseases, as well as the ideal dosage, is warranted. Leucine is an important amino acid that must be obtained from food and available from animal- and plant-based sources (Bauer et al., 2015). Some of the finest leucine is an important amino acid that must be supplied via food and may be found in animal- and plant-based sources (Bhat and Karim, 2009). Some of the finest leucine food sources include animal proteins are a source of leucine. Leucine is found in meat, chicken, fish, eggs, and dairy products. For example, three ounces of chicken breast or beef have around 1.5 g of leucine, whereas a cup of milk or yoghurt contains about 0.7 g (Gardner et al., 2019).Proteins from plants Leucine is also found in beans, lentils, peas, soy products, and whole grains. A cup of cooked lentils or soybeans, for example, has around 1.3 g of leucine, while a cup of cooked quinoa contains about 0.9 g. In addition, some food additives and preservatives may also affect the nutritional value of leucine and other amino acids a study found that the addition of sodium nitrite and sodium erythorbate to meat products resulted in a decrease in leucine content. The authors suggest that the reduction in leucine content may be due to the reaction between the preservatives and the amino acids present in the meat (Heaton et al., 2000). This reaction can lead to the formation of nitrosamines, which are known to have carcinogenic effects (Hecht, 1997). Additionally, the reduction in leucine content may have negative effects on muscle protein synthesis, which is important for muscle growth and maintenance (Deutz et al., 2011). Overall, while the current research on leucine is extensive, there are still many areas that need to be addressed in order to fully understand its role in regulating growth and development. Further research into the tissue-specific effects of leucine, its long-term impacts, optimal dosing, and interactions with other nutrients is needed to inform dietary recommendations and improve health outcomes.

### 1.1 Leucine transport and metabolisms

Leucine, being an indispensable amino acid, is not endogenously produced inside the human body, necessitating its acquisition through dietary sources. Following the ingestion of a meal containing protein, the content of leucine experiences a rapid increase. Subsequently, leucine is transported across the cell membrane through a group of amino acid transporters known as L-type amino acid transporters (LATs) (Donato et al., 2006). The family comprises four Na + -independent neutral amino acid transporters, namely, LAT1-LAT4. LAT1 and LAT2 are sometimes referred to as solute carrier (Slc) 7, specifically Slc7a5 and Slc7a8, respectively. Similarly, LAT3 and LAT4 are recognized as Slc43, namely, Slc43a1 and Slc43a2, respectively (Wang and Holst, 2015). LAT1 and LAT2 proteins exhibit a dependency on a binding partner and exhibit a broader spectrum of transport for neutral amino acids as compared to LAT3 and LAT4 proteins. The latter proteins function as facilitated diffusers and demonstrate a higher degree of specificity towards transporting leucine, isoleucine, valine, phenylalanine, and methionine (Kanai et al., 1998; Wang and Holst, 2015). The four transporters have distinct expression patterns and tissue localization, but with some degree of overlap (Segawa et al., 1999). LAT1, also known as Slc7a5, has been extensively investigated due to its prominent association with the spleen, activated lymphocytes, and brain (Mastroberardino et al., 1998). The binding of leucine to LAT1 has received particular attention in research. The transfer of leucine relies on glutamine and occurs through a two-step transport mechanism (Nicklin et al., 2009). Initially, it should be noted that glutamine is delivered into the cell through the utilization of the glutamine transporter (Slc1a5), which plays a crucial role in regulating the intracellular levels of glutamine. Subsequently, a molecular complex consisting of Slc7a5 and Slc3a2, which are both glutamine transporters, employs intracellular glutamine as an efflux substrate in order to modulate the cellular absorption of external leucine (Nicklin et al., 2009). Upon entering the cellular environment, leucine exhibits the ability to influence many cellular processes, become a constituent of protein structures, or initiate its breakdown process by transferring its amino group to a-ketoglutarate through a transamination reaction (Sweatt et al., 2004). The process of leucine breakdown largely takes place in the mitochondria of skeletal muscle and other tissues. This is because the liver has limited capacity for leucine, isoleucine, and valine transamination (Hutson et al., 1988). The enzyme BCATm catalyzes the process of leucine transamination. The process described involves the transfer of the a-amino group of leucine to-ketoisocaproate, which is a reversible reaction. Around 20% of the amino acid leucine undergoes conversion into ketoisocaproate (KIC), whereas the remaining portion of leucine is utilized for the process of protein synthesis inside skeletal muscle (Duan et al., 2016). In certain tissues, glutamate can undergo either amidation to form glutamine or transamination to produce a-ketoglutarate, resulting in the generation of alanine.

## 2 Regulation mechanisms and function of leucine

The process by which cells create new proteins from amino acids is known as protein synthesis (Argilés et al., 2016). It is essential for developing and maintaining all bodily tissues, including skeletal muscle. Skeletal muscle is very vital for mobility and accounts for a huge portion of the body’s protein turnover. As a result, protein synthesis is critical for sustaining muscle mass and function (Phillips et al., 2009). Protein synthesis in skeletal muscle is a complicated set of metabolic events controlled by many signaling channels. The mTOR pathway, which is stimulated by foods such as amino acids, particularly leucine, is one of the main routes. Leucine stimulates mTOR signaling, which increases muscle protein synthesis and promotes muscular development (Castro et al., 2016). The BCAAs stimulate this procedure by enhancing mTOR pathway components TSC2, Rheb, and Raptor at the onset of mRNA translation (Argueta et al., 2018). The mTOR (mammalian target of rapamycin) pathway regulates protein synthesis and cell proliferation. Upstream signaling molecules that modulate the mTOR pathway include tuberous sclerosis complex 2 (TSC2), a Ras homolog abundant in the brain (Rheb), and the mTOR regulatory-associated protein of mTOR complex 1 (Raptor). TSC2, Rheb, and Raptor are all important regulators of the mTOR pathway, which is a crucial regulator of protein synthesis and cell development. TSC2 inhibits Rheb, which inhibits mTOR activity, while Rheb promotes mTOR to boost protein synthesis. Raptor coordinates the activation and control of mTORC1 to begin protein synthesis in retorting to amino acids (Hong-Brown et al., 2012). The interaction of these molecules regulates the activity of the mTOR pathway, which regulates protein synthesis and cellular development (Hong-Brown et al., 2012). The mechanistic target of rapamycin complex 1 [mTORC1] and mechanistic target of mTOR complex 2 [mTORC2] make up the single MTOR gene in mammals as opposed to the two TOR genes (TOR1 and TOR2) found in yeast (Kim and Guan, 2019). mTOR (mammalian target of rapamycin) is a protein kinase that regulates several physiological functions such as protein synthesis, metabolism, cell growth and proliferation, autophagy, and immunology. mTOR is composed of two distinct complexes, mTORC1 and mTORC2. mTORC1 is the major complex involved in protein synthesis control; it is triggered by a variety of signals, including nutrients, growth hormones such as insulin, and amino acids such as BCAAs (branched-chain amino acids), particularly leucine (Wataya-Kaneda, 2015). mTORC1 regulates protein synthesis by phosphorylating and activating downstream substrates such as S6K (S6 kinase) and 4EBP1/2 (eIF4E-binding protein 1/2), influencing mRNA translation initiation. Rag GTPases are mTORC1 activators; they are part of a nutrient-sensing system that adjusts mTORC1 activity in response to amino acids such as leucine (Chong, 2015). Rag GTPases are defined as a “molecular switch” that activates mTORC1 when amino acids are abundant and deactivates it when they are sparse. mTORC2 has a structure similar to mTORC1, although it is not mainly involved in protein synthesis control. mTORC2 is instead known to regulate the cytoskeleton, differentiation, and cellular metabolism, including glucose and lipid metabolism (Proud, 2019). Both complexes exhibit unique elements and upstream regulators, resulting in downstream signaling variations. Mammalian LST8 [mLST8], proline-rich Akt substrate of 40 kDa [PRAS40], regulatory-associated protein of mTOR [Raptor], regulatory-associated protein of [mTOR], and Dishevelled, EGL-10, and pleckstrin [DEP] domain-containing mTOR make up mTORC1. Raptor is in charge of improving the recruitment of substrates to mTORC1 and is necessary for mTORC1’s localization to the lysosomal membrane (Maiese et al., 2013). mTORC2 contains components that are distinct from mTORC1, such as Raptor-independent companion of mTOR [Rictor] and mammalian stress-activated protein kinase interacting protein [mSin1], as well as shared components like mLST8 and DEPTOR (Awasthi et al., 2016).

**FIGURE 1:**
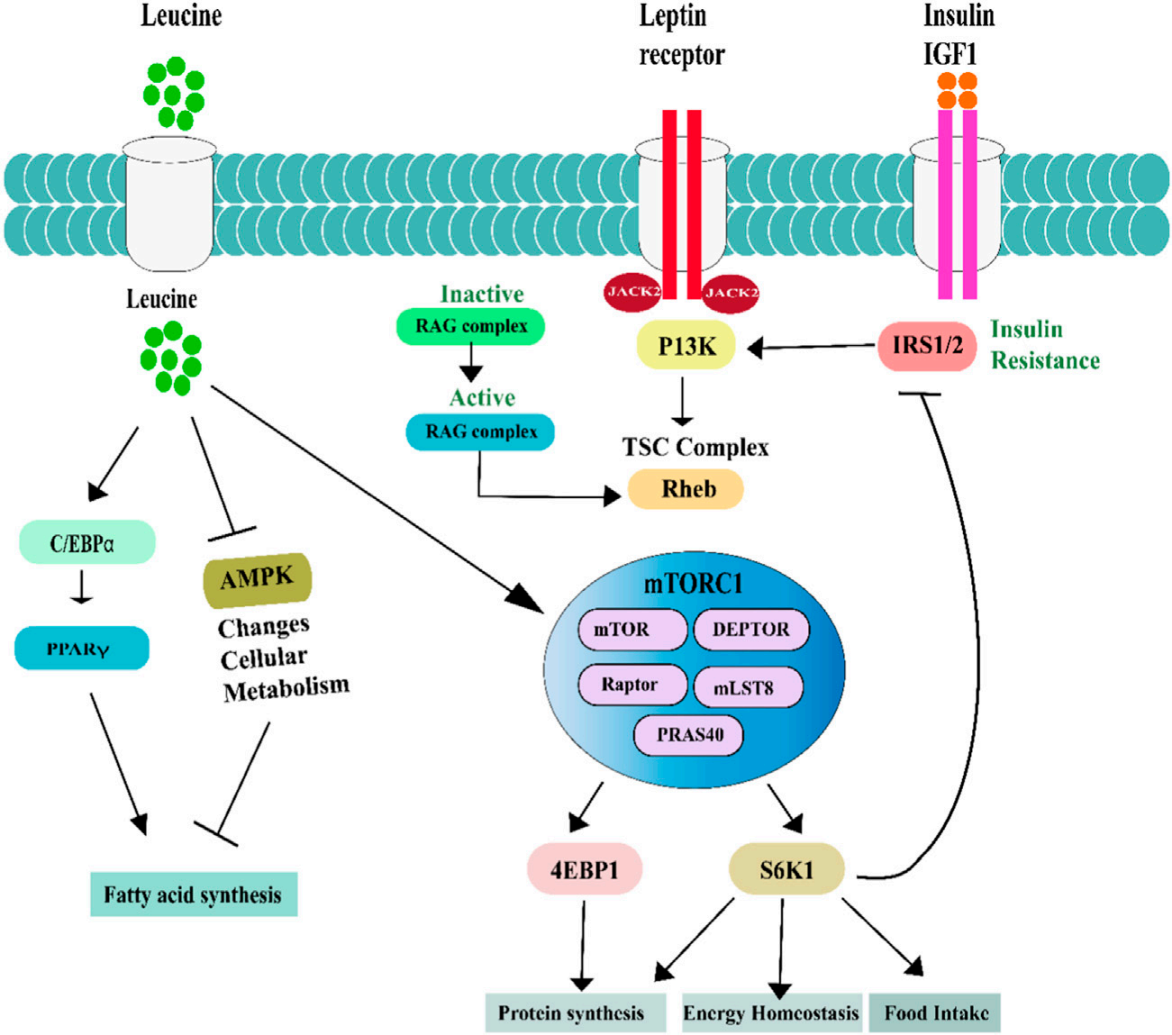
mTORC1 comprises the components mTOR, Raptor, mLST8, PRAS40, and DEPTOR. mTORC1 is activated by amino acids (especially Leucine) and hormones like leptin, insulin, and IGF-1. A multitude of ways may activate mTORC1. The TSC complex is critical in the activation of hormones. However, mTORC1 activation by amino acids requires the Rag complex. The p70-S6 kinase 1 (S6K1) and eukaryotic initiation factor 4E (eIF4E) binding protein one (4E-BP1) proteins are two of mTORC1’s major downstream targets. S6K1 phosphorylates components of the insulin signaling pathway when nutrients are abundant, as happens in obesity, leading to insulin resistance. The anorexigenic effects of leptin rely on two separate signaling pathways: mTOR/S6K1 and phosphatidylinositol-4,5-bisphosphate 3-kinase (PI3K). The quick initiation and persistence of leptin’s anorexigenic activities may need the PI3K/mTOR/S6K1 pathway since mTOR is a downstream target of PI3K signaling. Leucine activated the C/EBP that leads to PPARy occur Fatty acid synthesis.

While PRAS40 improves both mTORC1 and mTORC2 functions, DEPTOR suppresses both complexes. A more detailed examination of the structure and composition of mTOR complexes can be found elsewhere (Wiza et al., 2012). mTOR signaling was studied in the development of breast cancer and resistance to targeted therapy (Figure 1). The research discovered that mTOR signaling hyperactivation is linked to the development of breast cancer and resistance to targeted therapy (Dong et al., 2021). The researchers used mathematical modeling to investigate the signaling networks that govern mTOR hyperactivation in breast cancer cells. They discovered that oncogenic protein kinase C (PKC) activation plays a crucial role in the hyperactivation of mTOR signaling in breast cancer cells. PKC is an enzyme that controls cell growth, differentiation, and survival (Fyffe and Falasca, 2013) PKC overexpression has been linked to various malignancies, including breast cancer. Furthermore, scientists discovered that inhibiting PKC signaling lowered mTOR activity and cell growth and proliferation (Tee et al., 2003). The researchers also looked at the significance of mTOR signaling in breast cancer cell resistance to targeted therapy (O’Brien et al., 2014). They discovered that cancer cells resistant to targeted medicines like trastuzumab had greater levels of mTOR signaling activity than cells responsive to targeted therapies (Gajria and Chandarlapaty, 2011). In one of the studies, the researchers discovered that leucine supplementation boosted the phosphorylation of downstream targets of the mTOR pathway, such as ribosomal protein S6 and eukaryotic initiation factor 4E binding protein 1 (4E-BP1) in the *in vitro* section of the study. Protein synthesis genes such as eukaryotic elongation factor 2 and ribosomal protein S6 were upregulated in response to leucine supplementation in C2C12 myotubes (Salles et al., 2013). In this study, the researchers separated the male Sprague-Dawley rats into two groups: a control group and a leucine-supplemented group. The leucine-supplemented group received 2.25 g/kg body weight per day of leucine for 10 days (Zanchi et al., 2012). The phosphorylation levels of mTOR and downstream targets and protein production were assessed in rat skeletal muscle. The findings demonstrated that leucine supplementation increased the phosphorylation of mTOR and its downstream targets, including p70S6K and 4E-BP1, in the rats’ skeletal muscles. Increased phosphorylation was followed by increased protein synthesis in the rats’ skeletal muscles. The researchers also discovered that supplementing with leucine caused muscular hypertrophy, as indicated by an increase in cross-sectional area and muscle fiber diameter (Biolo et al., 1995). According to the findings, leucine supplementation increases the mTOR signaling system, resulting in increased protein synthesis and, as a result, muscular growth. The researchers hypothesized that leucine supplementation enhances amino acid availability in skeletal muscle, activating the mTOR signaling pathway. The study of Laplante and Sabatini provides a comprehensive description of how mTOR signaling regulates protein translation, metabolism, cell growth, proliferation, and survival by integrating external and intracellular inputs (Ben-Sahra and Manning, 2017). Tumor development, angiogenesis, insulin resistance, adipogenesis, and T-cell activation are all biological activities activating mTOR due to their high energy and food demands (Ananieva et al., 2016; Carbone et al., 2012). Mammalian Rag GTPases A-D may combine into heterodimers. Rag A and Rag B are a heterodimer that interacts with mTORC1 [via Raptor] and is activated by the amino acid leucine, which promotes Rag A’s and Rag B’s GTP loading (Sancak et al., 2008). For Leucine to perform its regulatory role, it must be ligated to its transfer RNA, which is catalyzed by the enzyme leucyl-transfer RNA synthetase (Wang X. et al., 2021). This enzyme responds to cellular levels of Leucine by stimulating the Rag complex. After being activated by Rag GTPase, mTORC1 is triggered to go to the surface of lysosomes (Bhat and Karim, 2009). That’s where mTORC1 activates itself by binding to another protein, the small GTPase Ras homolog abundant in the brain [Rheb] (Jewell et al., 2015). The lysosome plays a crucial role in amino acid sensing by the mTORC1 signaling pathway. The interaction between mTORC1, Rag GTPases, and Rheb on the lysosomal surface requires the presence of amino acids like Leucine (Greenwell et al., 2017). Moreover, a leucine-supplemented diet has enhanced IGF-1 and IGF-2 gene and protein expression in the fetal liver, potentially alleviating fetal growth constraints. One recent research shows that overexpression of Leucine in SLC6A14 increased blastocyst activation. mTOR is a key player in blastocyst development (González et al., 2012). The study shows that Leucine is important in regulating the mTORC1 signaling pathway in various cellular functions. Recent research has identified additional roles for leucine in various other physiological processes, including adipocyte differentiation, lipid metabolism, and bone metabolism (Ra and Dm, 2017). The mechanisms leucine regulates these processes are complex and involve multiple signaling pathways. Overall, the current research suggests that leucine supplementation may have therapeutic potential in treating various metabolic and musculoskeletal disorders. Furthermore, protein synthesis is necessary for general health and wellbeing. Proteins have several roles in the body, including the synthesis of enzymes, hormones, and neurotransmitters. They also give structural support to cells and tissues, aid in chemical movement throughout the body, and play a significant role in immunological function.

## 3 Regulation of protein synthesis through mTOR

Since 1999, the pioneer in leucine functional research has investigated Leucine’s impact on muscle protein synthesis and its mechanisms (Hecht, 1997). First, his team demonstrated that Leucine increases human muscle protein synthesis and recovery after exercise despite increasing plasma insulin levels (Anthony et al., 2000; Zhang et al., 2017). The mammalian target of rapamycin (mTOR) is a protein kinase, an enzyme that regulates cellular activities such as cell development, proliferation, and metabolism. mTOR is a key component of the signaling system that regulates muscle protein synthesis in response to leucine and other amino acids (Hall, 2008). These proteins are regulated by mTOR kinase activity and are called downstream target proteins. p70 ribosomal S6 kinase 1 (S6K1) and eukaryotic initiation factor 4E-binding protein 1 (4E-BP1) are two of the most important downstream target proteins of mTOR in skeletal muscle protein synthesis. The eukaryotic cell’s first growth factor, Protein 4E-binding protein 1 (4E-BP1), binds to the essential eukaryotic initiation factor 4E (eIF4E) and blocks its ability to initiate protein translation (Argilés et al., 2016). When leucine and other amino acids activate mTOR kinase, it phosphorylates 4E-BP1, causing it to relieve inhibition on eIF4E. This release enables the eIF4F complex to assemble, which is required for mRNA translation to start. P70 ribosomal S6 kinase 1 (S6K1) is a protein kinase that phosphorylates ribosomal protein S6 in response to leucine and other amino acid stimulation of mTOR. Ribosomal protein S6 is required for the translation of mRNA into new proteins. For this procedure to be effective, S6 must be phosphorylated by S6K1 (Deng et al., 2014). After many experimental results showed that Leucine stimulates muscle protein synthesis in pigs, rats, and humans (Crozier et al., 2005). One study found that for 4 weeks, the researchers gave male rats a diet containing varying doses of leucine. Leucine amounts evaluated were 3%, 4.5%, and 6% of total dietary protein, respectively (Li et al., 2013). The study revealed that increasing leucine consumption was linked to a dose-dependent increase in muscle protein synthesis rates (Robinson et al., 2013). The researchers discovered that rats with a diet containing 6% leucine had the best muscle protein synthesis rates. Furthermore, muscle protein synthesis rates rose by nearly 20% in rats with a 4.5% leucine diet compared to those with a 3% leucine diet (Norton et al., 2009). The authors hypothesized that the rise in muscle protein synthesis rates seen with increased leucine consumption was caused by the activation of muscle protein synthesis signaling pathways. Leucine, in particular, stimulates the mTOR signaling pathway, which is important in controlling muscle protein synthesis. Furthermore, according to the researchers, leucine enhanced the phosphorylation of the eukaryotic initiation factor 4E binding protein-1 (4E-BP1), which is involved in controlling protein translation initiation. This data implies that leucine may also improve protein translation efficiency, increasing muscle protein synthesis rates (Norton et al., 2009). Overall, the findings imply that increasing dietary leucine consumption may improve muscle protein synthesis rates in rats, likely via activation of the mTOR signaling pathway and higher protein translation efficiency (Li et al., 2013). Another study, especially in neonates, discovered that supplementing Leucine or its metabolites alpha-ketoisocaproic acid and B-hydroxy—methyl butyrate significantly increases newborn muscle protein synthesis (Wheatley et al., 2014). Protein synthesis regulators PHAS-I and p70 S6 kinase are upregulated in pancreatic -cells in response to leucine supplementation in a protein-deficient diet. These cells are responsible for protein translation and mitogenic signaling (Peng et al., 2012). Here are some studies in the table related to leucine help in growth development and protein synthesis.

Table 1 shows that leucine helps in muscle growth in different Animal models.

**TABLE 1:** The effect of Leucine on muscle growth in different animal models.

Animal model	Studies	Finding	References
Pigs	Vuppusetty C, et al.	Leucine supplementation improved lean body mass, reduced fat, and enhanced muscle protein synthesis.	Boultwood J, Yip B H, Vuppusetty C, et al. Activation of the mTOR pathway by the amino acid L-leucine in the 5q syndrome and other ribosomopathies[J]. Advances in biological regulation, 2013, 53(1): 8–17
Chickens	Nie, Cunxi, et al.	Leucine supplementation improved muscle growth and development, as well as feed efficiency.	Nie, Cunxi, et al. “Branched-chain amino acids: beyond nutrition metabolism.” *International Journal of molecular sciences* 19.4 (2018): 954
Mice	D'Antona et al.	Leucine supplementation increased muscle mass and improved muscle function, particularly in older mice.	D'Antona, G., et al. “Branched-chain amino acid supplementation promotes survival and supports cardiac and skeletal muscle mitochondrial biogenesis in middle-aged mice.” Cell metabolism 12.4 (2010): 362–372.
Zebrafish	Zhu, Qiang	Leucine supplementation improved skeletal muscle growth and development in zebrafish larvae.	Zhu, Qiang-Sheng, et al. “Early leucine programming on protein utilization and mTOR signaling by DNA methylation in zebrafish (*Danio rerio*).” *Nutrition and Metabolism* 17.1 (2020): 1–13

Leucine has been shown to increase MPS and even certain other physiological processes. This approach entails collecting a muscle biopsy from a patient before and after leucine administration, then analyzing the tissue’s protein level to determine leucine’s impact on MPS and other physiological processes. The findings will reveal whether or not MPS rises after taking leucine. The authors suggest Leucine may be critical for variable insulin secretion and glucose homeostasis (Yang et al., 2010). Leucine is more effective than other necessary amino acids in boosting MPS via activating mTORC1 (Le Plénier et al., 2012). Leucine also reduces muscle proteolysis when paired with exercise, resulting in a synergistic MPS response. Activation of mTORC1 and the operation of its “coincidence detector,” both controlled by small GTPases, are, thus, essential for protein synthesis in skeletal muscle (Reiners et al., 2022). One study examined the effects of leucine supplementation on lactating dairy cows. For 4 weeks, eight multiparous Holstein cows were fed a regular diet and supplemented with 100 g/day of leucine (the treatment group) or an equal amount of a placebo supplement (control group) (Schilde et al., 2021). The study’s findings revealed that cows in the leucine supplementation group produced considerably more milk than those in the control group, with an average increase of 2.2 kg, or 7.6%, more milk produced daily. Furthermore, the leucine supplementation group had considerably greater milk protein output and enhanced nitrogen use efficiency, indicating increased protein synthesis in the mammary gland (Yoder et al., 2020). Furthermore, the Leucine supplementation group had higher levels of growth hormone, insulin-like growth factor-I (IGF-I), and key signaling proteins such as mTOR and phosphorylated ribosomal protein S6, all of which are involved in normal mammary gland development and protein synthesis (Sobolewska et al., 2009). Another study discovered that supplementing with leucine might promote mammary gland growth and boost milk supply and composition in dairy cows. The study utilized 16 nursing Holstein cows randomly allocated to either a control or a treatment group and fed a diet supplemented with 60 g of leucine per day for 12 weeks. So cows in the leucine treatment group produced considerably more milk protein, fat, and lactose than the control group, with an average of 12.5% more milk produced daily (Gao et al., 2015). The study’s results suggest that supplementing the diet of nursing Holstein cows with Leucine can increase milk production and quality. Furthermore, the leucine supplementation group showed higher growth hormone, IGF-I, and mTOR signaling proteins in the mammary gland, indicating that protein synthesis and development were promoted (Gao et al., 2015). Leucine may have comparable impacts on other animal metabolic processes, including protein synthesis and muscle growth and development. Leucine supplementation may benefit animals with muscle-wasting disorders or people with sarcopenia, categorized by a loss of muscle mass and function. Furthermore, leucine supplementation may have used in sports nutrition as a dietary supplement for athletes wanting to increase muscle growth and improve performance (Holeček, 2017). The capacity of leucine to enhance muscle protein synthesis and growth offers athletes a viable technique for increasing lean muscle mass while decreasing recovery time after exercise. Similarly, a study found that leucine supplementation improved milk production and composition in lactating dairy cows by stimulating protein synthesis and mammary gland development (Li et al., 2011). Leucine has been shown to regulate animal growth and development through other mechanisms in addition to its effects on mTOR signaling. Supplementing with Leucine, for example, has increased the expression of genes in skeletal muscle, liver, and adipose tissue involved in energy and lipid metabolism (Surh et al., 2005). The studies looking into how leucine supplementation affects gene expression in fatty tissue, liver, and skeletal muscle. Male Wistar rats were given a diet with either 2% leucine or 1% leucine (the control group) for a period of 4 weeks (Donato et al., 2006). Supplementing with leucine significantly increased the expression of genes involved in energy and lipid metabolism in skeletal muscle, liver, and adipose tissue, as shown by the results of the research. The leucine supplementation group showed increased expression of genes involved in fatty acid oxidation, including carnitine palmitoyl transferase 1 (CPT1) and acyl-CoA oxidase (ACO), and genes involved in glucose metabolism, including glucokinase (GK) and glucose transporter 4 (GLUT4) (Donato et al., 2007). Leucine-supplemented rats showed greater amounts of transcription factors that regulate energy and lipid metabolism, such as peroxisome proliferator-activated receptor (PPAR) and insulin receptor substrate 1 (IRS1) (Eller et al., 2013). These alterations in gene expression were associated with higher insulin sensitivity and lower blood glucose and cholesterol levels, suggesting that leucine supplementation enhanced metabolic health (Yoon, 2016). Another study discovered the impact of leucine supplementation on gene expression in dairy goat liver and skeletal muscle. For 12 weeks, the goats were fed a diet enriched with either 0 or 0.80% leucine (Strable and Ntambi, 2010). So, the result in the liver and skeletal muscle, leucine supplementation increased the expression of genes involved in lipid metabolism, such as PPAR, acetyl-CoA carboxylase (ACC), and fatty acid synthase (FAS), as well as genes involved in glucose metabolism, such as glycogen synthase (GYS) and glucose transporter 1 (GLUT1). The goats given leucine also exhibited superior lipid profiles, increased glycogen stores in the liver, and enhanced insulin sensitivity (Zhao et al., 2020). Furthermore, Leucine administration boosted the expression of glucose metabolism genes such as glycogen synthase (GYS) and glucose transporter 1 (GLUT1) in the goats’ liver and skeletal muscle. These findings imply that Leucine supplementation may enhance glucose and lipid metabolism in dairy goats, perhaps leading to improved metabolic health and milk output. Leucine may also have anti-inflammatory and antioxidant effects that promote animal health and growth (Jansson et al., 2012). How adding leucine to a diet helps mice with NAFLD by altering their gene expression. When fat builds up in the liver without alcohol, a condition known as nonalcoholic fatty liver disease (Gao et al., 2019), the researchers utilized mice fed a high-fat diet to generate NAFLD. The mice were then separated into two groups, one getting leucine supplementation (at a dosage of 0.68% leucine) and the other serving as a control group with no supplementation. The research lasted 8 weeks (Kim B. et al., 2017). The study’s findings revealed that leucine supplementation dramatically boosted the expression of genes involved in lipid metabolism in the livers of NAFLD mice. Leucine-supplemented mice showed higher levels of expression of PPAR and acyl-CoA oxidase (ACO), both of which are involved in the oxidation of fatty acids in the liver (Landon et al., 2020). In a separate experiment, researchers examined how leucine supplementation affected gene expression in the skeletal muscle of diabetic rats. The rats were given a high-fat diet with either no added leucine (control group) or 0.5% leucine (test group) for 8 weeks. Leucine supplementation significantly increased the expression of genes involved in glucose and lipid metabolism in skeletal muscle, including GLUT4, peroxisome proliferator-activated receptor (PPAR), and lipoprotein lipase (LPL). Leucine supplementation improved glucose tolerance, insulin sensitivity, and muscle structure in diabetic rats. These results suggest that leucine supplementation may prevent and cure type 2 diabetes by increasing insulin sensitivity and regulating gene expression in glucose and lipid metabolism (Zhang et al., 2007). The above studies show that leucine supplementation can boost the expression of genes complicated in energy and fat metabolism in a variety of tissues. This shows that leucine might be useful in treating metabolic illnesses such as diabetes, obesity, and fatty liver disease, which are characterized by dysregulation of energy and lipid metabolism. Generally, this study provides evidence that Leucine activates the mTORC1 pathway through a complex molecular mechanism involving the translocation of mTOR to the lysosomal surface and the activation of downstream effectors. Leucine’s activation of the mTORC1 pathway is a crucial mechanism by which Leucine promotes protein synthesis and muscle growth.

## 4 Mechanism of leucine in animal growth and development

The capacity of leucine to activate the mechanistic target of the rapamycin (mTOR) signaling pathway, which plays a key role in controlling protein synthesis and cell proliferation, is well established. (Kimball and Jefferson, 2006). Recent research has given insight into the intricate processes involved in the leucine sensing system, which underpins mTOR activation. Sestrin2, CASTOR1, and leucyl-tRNA synthetase (LRS) are the three primary components of the leucine sensing system (Sekine et al., 2021). Here’s some background on each component and its involvement in the leucine sensing system (Schuler et al., 2021). Sestrin2 is a stress-responsive protein that is essential for the leucine sensing system. Sestrin2 has been reported to interact with many proteins in the pathway, including GATOR1 and GATOR2, which control the activities of mTORC1 via the Rag GTPases. Sestrin2 can activate mTORC1 indirectly by blocking GATOR2 or activating GATOR1, enabling Rag GTPases to activate mTORC1 (Ho et al., 2016). CASTOR1, a cytosolic protein, was recently shown to be an arginine sensor. CASTOR1 interacts with Sestrin2, which is required for mTORC1 activation in response to leucine. CASTOR1 can bind to leucine and inhibit GATOR2, activating mTORC1. LRS (leucyl-tRNA synthetase) is an enzyme that charges tRNA molecules with leucine (Chantranupong et al., 2016). According to recent research, LRS is also implicated in the leucine sensing system. LRS can interact with GATOR2 and decrease its function, resulting in mTORC1 activation. These three components collaborate to activate mTORC1 in response to leucine levels. The precise methods of interacting with and regulating one another are still being discovered (Liu and Sabatini, 2020). Understanding these pathways might greatly impact the development of novel medications and treatments for illnesses including cancer, metabolic disorders, and muscle wasting caused by mTOR signaling failure (Kim JH. et al., 2017). Activation of mTOR by leucine results in the stimulation of protein synthesis and the inhibition of protein degradation, leading to an overall increase in muscle mass (Norton and Layman, 2006). The insulin signaling system, which controls glucose metabolism and energy balance, is activated by Leucine in addition to the mTOR pathway. They used a mouse model to examine the effect of leucine on protein metabolism. They specifically looked at the effects of a low-protein diet on muscle mass and protein synthesis, as well as the ability of leucine supplementation to reverse these effects (Crozier et al., 2005). The mice were separated into four groups and fed various diets: a regular protein diet (20% protein), a low protein diet (5% protein), and a low protein diet supplemented with either 2% or 4% leucine. After 21 days, the researchers examined muscle mass, protein synthesis rates, and other protein metabolism markers (Zhang et al., 2007). The results revealed that mice fed a reduced protein diet had considerably less muscle mass than mice fed a regular protein diet. On the other hand, Mice fed a low-protein diet supplemented with leucine had more muscle mass than mice on a low-protein diet alone (Anthony et al., 2000). Furthermore, leucine supplementation enhanced protein synthesis rates in these mice’s muscles, demonstrating that leucine can have an anabolic impact on protein-restricted diets. By translocating glucose transporter type 4 (GLUT4) to the cell membrane, leucine-mediated activation of the AMPK pathway promotes glucose uptake in peripheral tissues, notably skeletal muscle. This mechanism promotes glucose absorption by cells and activates glycolysis and glucose oxidation for energy production (Farooqui, 2013). Enhancing both insulin secretion and glucose uptake and utilization by peripheral tissues, which regulates energy balance by promoting energy expenditure and inhibiting energy storage (Rieu et al., 2004). In one study, modest dosages of Leucine supplementation increased fat loss and efficiently boosted muscle protein synthesis in food-restricted rats (Rieu et al., 2004). Myofibrillar muscle protein synthesis in males can be stimulated by a low-protein (6.25 g) mixed macronutrient beverage including a high-protein (5 g total Leu) dosage (Churchward-Venne et al., 2014). In 1989 researchers looked at how men and women’s paths diverged. Fasting plasma amino acid levels were measured in 22 young men (aged 25–35), 21 older men (aged 65–85), 23 men (aged 65–92) with dementia who were fed orally in an institution, and 17 men (aged 65–88) who were fed artificially (Rudman et al., 1989). BCAA levels were highest in the first group, dropping in the third and fourth. In another research, 72 healthy volunteers aged 23 to 92 were split into six groups of 12 based on sex and age (41–42, 43–44, and >60 years). Men had higher amounts of essential and branched-chain amino acids (BCAAs) than women; total amino acid, EAA, and non-essential amino acid levels decreased with age (Clemmesen et al., 2000). A recent Korean study in adults aged 50–64 investigated the link between amino acid intake and skeletal muscle mass index (SMI). Leucine, isoleucine, and valine intake [measured by a 24-h recall] were all within the reference range for the Korean population, and there was a statistically significant positive correlation between BCAA intake [in g/day] with food and the SMI (Kang et al., 2020). The results of the aforementioned research indicate that supplementing with a leucine-rich protein may have positive benefits by halting muscle loss, especially among middle-aged men and women. Skeletal muscle recovery after exercise is also facilitated through leucine supplementation (Draganidis et al., 2017). So, you see the above data in different studies shows that BCAAs, especially Leucine, are essential for protein synthesis and growth development. Still, the actual age/Gram in the diet is required for further study.

### 4.1 Leucine’s role in growth metabolisms and adipocytes

We now know that mitochondrial depletion or dysfunction is linked to metabolic diseases (Zhao et al., 2022). Mitochondrial biogenesis plays a role in mediating the effects of Leucine on adipocyte lipid metabolism in favor of lipid partition to skeletal muscle (Duan et al., 2016). Leucine is essential for energy metabolism in adipocytes and skeletal muscles (Yin, 2015). Therefore, lipid metabolism provides the energy for Leucine-induced protein synthesis in skeletal muscle. Leucine is the only BCAA shown to affect skeletal muscle fatty acid oxidation, which is linked to mitochondrial biogenesis. Leucine, thus, increases mitochondrial bulk and oxygen consumption in both myocytes and adipocytes (Wen et al., 2019). These findings enhance the growing evidence supporting Leucine’s role in regulating energy expenditure. Excess adipose tissue produces chemicals that inhibit mitochondrial biogenesis, reducing fatty acid oxidation in skeletal muscle and metabolism of Fatty acid through Leucine (Dong et al., 2021). While Leucine is not directly involved in the metabolism of fatty acids, it can indirectly affect lipid metabolism by regulating protein synthesis and insulin signaling (Ye et al., 2020). Skeletal muscle is essential for keeping the body’s energy level stable because it is a significant site for clearing serum-free fatty acids [FFA] (Cui et al., 2017). A large body of research on the use of fatty acids and other lipids shows that Leucine controls the lipid metabolism of adipocytes (Duan et al., 2018). In one study, the experiment lasted 10 weeks, during which time the researcher fed 24 overweight women a regulated diet containing either high or low quantities of leucine. The high-leucine diet consumed 8.2 g of leucine daily, whereas the low-leucine diet consumed just 1.91 g. All diets had the same number of calories and macronutrients. Calorie and macronutrient levels were standardized across all diets (Layman, 2003). According to the study’s findings, compared to the low-leucine diet, the high-leucine diet increased FFA release by 58% due to its stimulation of lipolysis in adipose tissue. Weight, BMI, and waist circumference were all lower in the high-leucine diet group than in the low-leucine group, suggesting a potential function for leucine in weight reduction (Verreijen et al., 2015). Our understanding of how leucine increases lipolysis in adipose tissue is a gap. Previous studies have shown, however, that leucine may stimulate the mTOR pathway, which is essential in controlling lipolysis and other aspects of lipid metabolism. Leucine may also increase the expression of genes responsible for fatty acid oxidation, breaking lipids into energy (Lee et al., 2017). This increases the flow of free fatty acids [FFA] to the skeletal muscle, where they are used as energy substrates to fuel protein synthesis (SPRIET, 2002; Rudman et al., 1989). In addition to protein metabolism, HMB may also control lipid metabolism. HMB resulted in a 30% increase in the oxidation of the FA palmitate. Body fat decreased by 1.1 percent compared to 0.5% in the placebo group, and lean body mass increased by 1.4 kg compared to 0.9 kg (Rossi et al., 2017). As a result, there is an increase in lipid availability and plasma FFA concentration. There is no change in body mass despite increased lipolysis and decreased adipose tissue content (Herrera and Amusquivar, 2000). When FFA oxidation is improved, glucose use decreases, and increasing muscle mass is an excellent way to improve FFA oxidation. (Piatti et al., 1994). In one of the studies, after 6 weeks of dietary restriction, rats given a diet containing 1.7% leucine had 47% less body fat. Protein nutritional status and protein synthesis capability, on the other hand, rose (Campbell et al., 2001). These data suggest chronic low-dose leucine supplementation enhances fat loss, liver protein position, and muscle protein synthesis capacity in malnourished rats. Results showed that rats fed a diet containing 1.5% L-leucine had a 25% reduction in adiposity (Leucine, 2012). The oxidation of fatty acids in muscle cells was reduced by 62% when co-cultured with adipocytes or incubated for 48 h with adipocyte-conditioned media. Still, this impact was mitigated by treating adipocytes with Leucine (Li et al., 2022). So, this study suggests Leucine can coordinately boost energy partitioning from adipocytes to muscle cells, resulting in less energy stored in adipocytes and more fatty acids [FA] being utilized by muscle. The outcomes further support earlier research’s conclusions that leucine supplementation encourages body fat reduction. Leucine’s function in fat oxidation and mitochondrial biogenesis, therefore, may have an impact on mediating proper energy metabolism.

### 4.2 Metabolisms of leucine in lipid mitochondria biogenesis and insulin

The mitochondria are involved in both the lipid and energy metabolism of adipocytes. Muscle and adipose cells rely on mitochondria for energy metabolism, and this process incorporates Leucine as a mediator (Sun and Zemel, 2009). Mitochondrial genes such as peroxisome proliferator-activated receptor gamma co-activator 1-alpha [PGC-1 alpha] and silent information regulator transcript 1 [SIRT-1] may influence energy metabolism by controlling the mitochondrial number and fatty acid oxidation (Puigserver and Spiegelman, 2003). Leucine [0.5 mM] can control skeletal muscle and stimulate mitochondrial biogenesis in C2C12 myocytes and 3T3-L1 adipocytes. Energy metabolism is influenced in part by expression regulation. SIRT-1 and PGC-1 alpha (Sun and Zemel, 2009). Several *in vitro* and *in vivo* studies have revealed that Leu regulates lipid metabolism, mainly by slowing fat production and the breakdown and increasing energy consumption.

**FIGURE 2:**
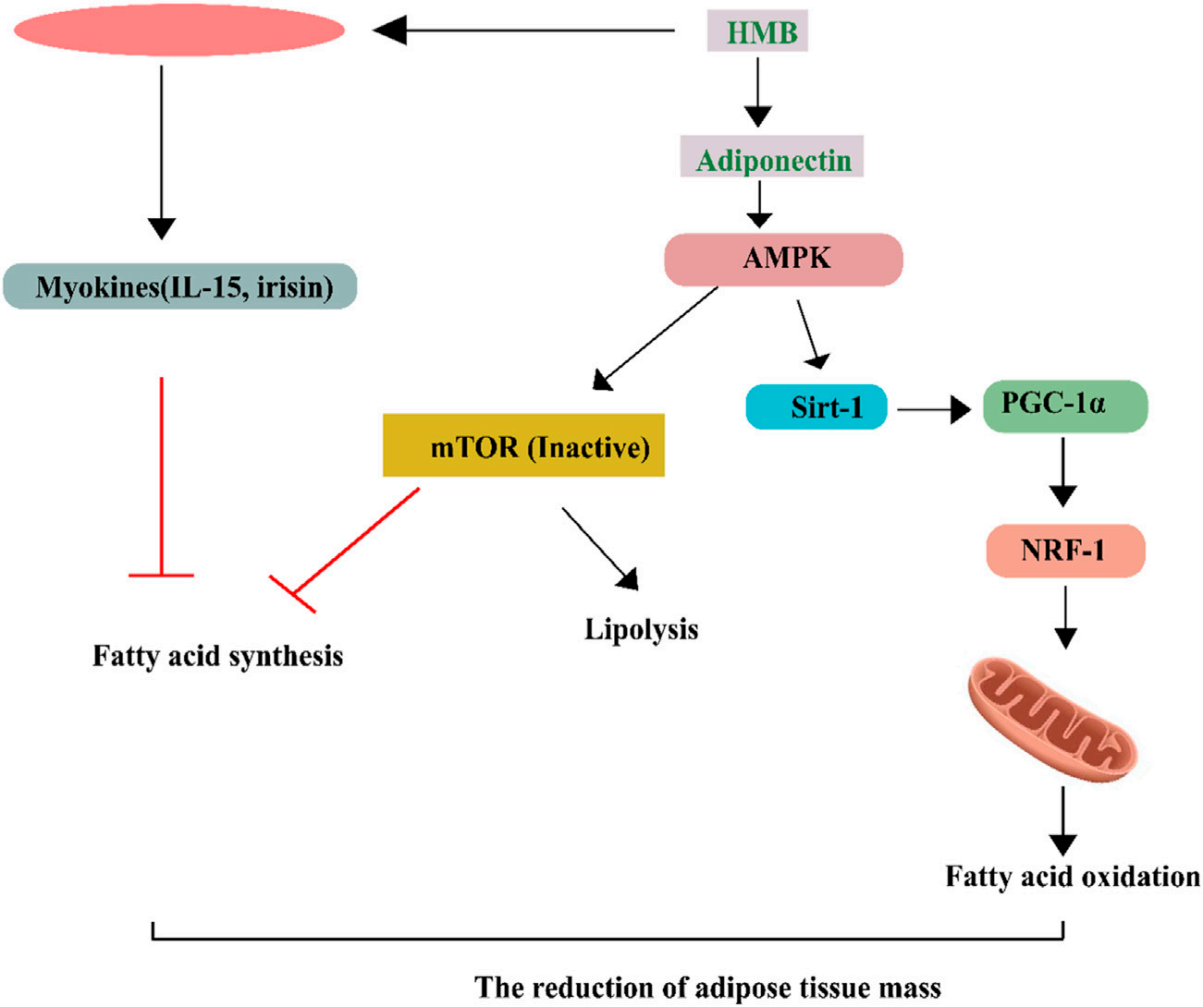
Leucine effects on lipid metabolism in adipose tissues, Skeletal muscle FAO and mitochondrial biogenesis are controlled by Leu’s activation of SIRT1, which in turn phosphorylates AMPK. Synergistic stimulation of the AMPK-SIRT1 pathway is achieved when Leu is combined with a modest dosage of metformin, which decreases the activation energy of NAD, induces PGC-1 phosphorylation and activation, and increases mitochondrial biogenesis and fatty acid oxidation (FAO). Activation of SIRT1-AMPK signaling promotes fatty acid oxidation and suppresses lipogenesis via deacetylating and phosphorylating PGC-1, respectively.

Moreover, activation of AMP-activated protein kinase [AMPK] causes phosphorylation and enhanced activity of PGC-1 alpha, boosting mitochondrial function (Zhang et al., 2020). Leucine’s effects on insulin signaling are believed to be mediated by activating the mTOR signaling pathway. mTOR is a serine/threonine kinase that regulates cell growth, proliferation, and survival by controlling protein synthesis and metabolism. Leucine stimulates mTOR signaling by activating the Rag GTPases, which in turn activate mTOR complex 1 [mTORC1] and promote the phosphorylation of downstream targets such as S6 kinase 1 [S6K1] and eukaryotic initiation factor 4E-binding protein 1 [4E-BP1] (Figure 2) (Catania et al., 2011). The activation of mTOR signaling by Leucine is thought to enhance insulin signaling by promoting the synthesis of insulin receptor substrate 1 [IRS-1] and Akt [protein kinase B], which are vital components of the insulin signaling pathway (Greenwell et al., 2017). Activated mTORC1 promotes the translation of IRS-1, a major component of the insulin signaling pathway required for activating insulin receptors and downstream signaling. When insulin activates it, the insulin receptor phosphorylates IRS-1, which activates downstream targets. Insulin phosphorylation activates Akt, a crucial insulin signaling pathway downstream target. Akt activation is the root cause of increased glucose absorption and storage in skeletal muscle, liver, and adipose tissue. Moreover, mTORC1 activation by Leucine may enhance glucose uptake and metabolism in skeletal muscle and other insulin-sensitive tissues (Takano et al., 2001). Leucine has also been shown to help muscle cells use insulin better and take in more glucose. Leucine supplementation’s effect on glucose uptake in healthy young males was studied. In a double-blind, randomized, crossover design, eight healthy male participants were given a leucine supplement (6 g of leucine mixed with water) or a placebo (Smith et al., 2011a). Following a 12-h fast, participants were told to ingest either the leucine supplement or a placebo before a glucose tolerance test. To measure glucose absorption, skeletal muscle samples were obtained at baseline and 45 min following the glucose tolerance test (Longo and Mattson, 2012). Leucine supplementation increased glucose absorption in skeletal muscle, according to the findings. In particular, compared to the placebo, glucose absorption by skeletal muscle tissue increased by roughly 25% following leucine administration (Smith et al., 2011b). The authors postulated that supplementing with leucine improved glucose absorption by activating the mTORC1 pathway, stimulating protein synthesis and cell development in skeletal muscle. They hypothesized that the enhanced glucose absorption helped the subjects improve their glucose utilization and insulin sensitivity (Liu et al., 2009). Overall, this study provides evidence to support the role of Leucine in promoting glucose uptake in skeletal muscle, suggesting that Leucine supplementation may be a valuable strategy for improving metabolic function in healthy individuals. The effects of leucine supplementation on insulin sensitivity in overweight and obese individuals were studied. Prior to a liquid meal challenge, 14 overweight or obese men were randomly randomized to consume either a leucine-supplemented drink (200 mg/kg body weight of leucine) or a placebo drink (Liu et al., 2009). The study’s findings revealed that leucine supplementation increased insulin sensitivity in individuals by roughly 28% as compared to the placebo group. Participants who took the leucine supplement also had lower fasting insulin levels and a better lipid profile than those who took the placebo (Ghorbani et al., 2023). The authors proposed that supplementing with leucine may increase insulin sensitivity by activating the mTORC1 pathway, which can drive protein synthesis and cell growth. They also mentioned that leucine may have anti-inflammatory characteristics, which might contribute to the observed improvements in insulin sensitivity. This may be good for lipid metabolism (Giron et al., 2022). Overall, while there is limited research on the direct effects of Leucine on fatty acid metabolism, leucine supplementation may be a valuable strategy for improving body composition and metabolic health through its effects on protein synthesis and insulin signaling.

### 4.3 mTOR and AMPK regulate energy in muscle

Cellular stress can turn on AMPK, the primary energy sensor in cells, and cause intracellular AMP to rise (Wu et al., 2014). AMP-activated protein kinase is essential for energy consumption, cell proliferation, and cell differentiation. Coenzyme inactivity Carboxylase activity activates AMPK, which boosts fat oxidation, lowers glucose generation, affects cholesterol and total triglyceride formation, disrupts mTOR signaling, and inhibits lipogenesis (Xin et al., 2016). Leucine performs a unique role in energy metabolism by circuitously activating AMPK to increase leptin and adiponectin secretion and synthesis from adipocytes (Zhang et al., 2020). The cell’s primary energy sink is the process of making proteins. mRNA translation and ribosome biogenesis require a lot of energy, and mTOR pathways significantly affect both processes (Dobrenel et al., 2016). When cells lack either an AA substrate or adequate energy, they slow their rate of protein synthesis through an mTOR-dependent process (Liu and Sabatini, 2020). This has led researchers to hypothesize that mTOR activity is related to the amount of energy available to cells.

#### 4.3.1 Leucine AMPK and obesity

Obesity and diabetes are treatable and preventable conditions that may benefit from dietary interventions. The mTOR signaling pathway is essential for brain systems that adjust energy expenditure in response to changes in nutritional supply (Cetinkalp et al., 2014). The leucine loop inhibits insulin signaling and reduces glucose consumption in skeletal muscle by activating mTOR and S6K1(8). Both normal rats and ob/ob mice had less appetite and lower body weight [BW] after taking leucine. It is comparable to the results of a high-protein diet, suggesting that Leucine plays a significant role in those results. Investigated the effect of Leucine supplementation on food intake and body weight in rats. In the study, two groups of rats had diets containing either 9% or 36% Leucine by weight. After 14 days, the rats with the high Leucine diet consumed less food and experienced an 11% reduction in body weight compared to the control group (Lynch et al., 2006). Moreover, the study showed that Leucine supplementation increased lean body mass while reducing animal fat mass. The researchers concluded that Leucine’s effects on food intake, body weight, and body composition may be due to its impact on regulating energy balance within the body (McAllan et al., 2013). Leucine and high protein diets also decrease AMP-activated protein kinase [AMPK] activity in the hypothalamus while increasing mTOR activity, inhibiting NPY, and stimulating proopiomelanocortin production (Freudenberg et al., 2012). Several additional signaling mechanisms control the mTOR system, including AMP-activated protein kinase (AMPK) and neuropeptide Y (NPY). AMPK is a cellular energy sensor that governs cell metabolism and regulates energy balance. It suppresses mTOR signaling in low-energy situations, such as fasting or exercise, which may interfere with protein synthesis. In the hypothalamus, NPY is a neurotransmitter that controls hunger and energy balance. NPY signaling also regulates mTOR activity in peripheral tissues such as skeletal muscle (Mobley et al., 2015). Unlike AMPK, NPY increases protein synthesis in skeletal muscle by activating mTOR signaling. On the other hand, NPY signaling counteracts the effects of AMPK and promotes mTOR signaling and protein synthesis. In turn, this can enhance the effects of leucine on protein synthesis and skeletal muscle growth. The researchers used cultured skeletal muscle cells from rats to test this theory (Norton et al., 2012). They initially treated the cells with NPY, AMPK activators, or AMPK inhibitors and then assessed the activity of mTOR signaling and protein synthesis. They discovered that NPY boosted the activity of mTOR signaling and protein synthesis, while AMPK activators inhibited these cellular activities. In contrast, AMPK inhibitors boosted mTOR signaling and protein synthesis (Norton et al., 2012). The researchers next investigated the interplay of the NPY and AMPK signaling pathways and their impact on mTOR signaling and protein synthesis. They discovered that NPY signaling counteracted the inhibitory effects of AMPK activators on mTOR signaling and protein synthesis. Specifically, AMPK activators reduced phosphorylated mTOR and protein synthesis, but both levels were recovered when paired with NPY therapy (Cota et al., 2007). The researchers also looked at the molecular processes behind the NPY and AMPK signaling pathways interplay. They discovered that NPY stimulated the PI3K/AKT pathway, an upstream regulator of mTOR signaling. Furthermore, NPY administration decreased the activation of AMPK in skeletal muscle cells (Wang et al., 2022). Finally, the researchers conducted *in vivo* trials on rats to corroborate their results in cultured muscle cells. They injected NPY and AMPK activators into rat gastrocnemius muscles and assessed mTOR signaling and protein synthesis. Consistent with their results in cultured muscle cells, they discovered that NPY boosted mTOR signaling and protein production while counteracting the inhibitory effects of AMPK activators (Ramesh et al., 2017). There appears to be a leucine-dependent feedback loop involving AMPK and mTOR that controls eating. Furthermore, in their study, Adipocyte-derived hormones’ effects on peripheral tissue fatty acid oxidation may be modulated by AMPK; Leptin can activate AMPK in muscle, which is necessary for regulating energy homeostasis so the researchers investigated the effect of obesity on regional skeletal muscle and adipose AA metabolism using a combination of stable isotope tracers and arteriovenous balancing techniques (Tomé et al., 2021). Leucine is thought to help regulate blood sugar levels and enhance insulin sensitivity, which may contribute to improved glucose control and weight management. Leucine has been demonstrated in studies to boost the production of satiety hormones, which may lower food intake and result in weight reduction (Gannon et al., 2003). One study published examined leucine’s impact on healthy individuals’ hunger and food consumption, Before a standardized breakfast, 24 individuals were given either a leucine-enriched amino acid drink or a control drink (Acid et al., 2023). The researchers discovered that individuals who drank the leucine-enriched beverage had higher levels of satiety hormones such as glucagon-like peptide-1 (GLP-1) and peptide YY (PYY) than those who drank the control beverage. These hormones have been shown to decrease food intake while increasing feelings of fullness (Blomstrand et al., 2006). Furthermore, individuals who drank the leucine-enriched beverage had lower levels of ghrelin, a hormone that raises hunger and encourages food consumption. Consequently, those who drank the leucine-enriched drink ate fewer calories at their next meal (Reinbach et al., 2009). Overweight and obese people were given a leucine-enriched amino acid supplement twice daily for 4 weeks in another study published in the journal Diabetes, Obesity, and Metabolism. The research discovered that individuals who took the supplement had lower hunger and food consumption than those who did not (Reinbach et al., 2009). These studies demonstrate that leucine may boost satiety hormones while decreasing appetite, resulting in lower food consumption and perhaps facilitating weight reduction. More study is required, however, to assess the long-term benefits of leucine supplementation on weight reduction and hunger management (Long and Zierath, 2006). Skeletal muscle contributes relatively little to the rapid rise of systemic leucine in obese people (Li et al., 2011). The majority of research has been conducted on small experimental animals such as rats or mice. To investigate the effectiveness of Leucine as a weight-loss therapy for obese people, long-term Leucine supplementation controlled meals, significant human volunteers, and confirmation by repetition are necessary.

Nie et al. (2018) Furthermore, Leucine improved glucose tolerance and insulin sensitivity in the animals. These results are consistent with other studies showing that Leucine supplementation may improve glucose metabolism and insulin sensitivity, which are critical factors in weight management and glucose regulation. The study suggests that Leucine supplementation may have several health benefits, including its effects on food consumption, body weight, and glucose regulation. However, further research is needed to explain the underlying mechanisms of Leucine’s effects on these factors and to determine if these benefits extend to humans.

## 5 Leucine zipper and gene growth regulation

The leucine zipper motif, which comprises a heptad repeating sequence of leucine residues, creates a protein dimerization domain that allows transcription factors to bind to DNA. Once bound, these factors may activate or repress gene expression by recruiting co-activators or co-repressors to the regulatory domains of target genes, respectively (Landschulz et al., 1988). This process is required for cellular differentiation, development, response to external stimuli, and preservation of cell homeostasis. The leucine zipper is a structural motif found in some transcription factors, which are proteins that regulate gene expression (Nijhawan et al., 2008). Within transcription factors, the leucine zipper motif generates an alpha-helical structure with a repeating heptad sequence of leucine residues that protrude from one face of the helix (Ogata et al., 1992). In every seventh position along the alpha helix, leucine residues face inward towards the helix center. When two transcription factors with leucine zipper motifs engage, their helices align, and the leucine residues of each factor interact in a complementary manner (Ellenberger et al., 1992). This contact between the two helices’ leucine residues is known as a “leucine zipper,” which creates a stable dimerization domain between the two transcription factors (Fang et al., 2018). The leucine zipper motif is a dimerization domain that allows two identical or similar proteins to bind together and form a functional transcription factor complex. One example of a gene regulated by a leucine zipper transcription factor is the c-fos proto-oncogene (Chang et al., 1990). One study discovered the significance of GCNF (Germ Cell Nuclear Factor), a nuclear hormone receptor superfamily member, in controlling gene expression throughout embryonic development. The researchers wanted to figure out how GCNF works in the transcriptional regulation of genes that affect cellular differentiation and development. The rest of the transcription factor can bind to particular DNA sequences and influence gene expression after the dimerization domain is established. The dimerization domain is required for transcription factor binding to DNA because it connects two DNA-binding domains, allowing them to interact collaboratively and with great specificity (Rajković et al., 2010). The researchers investigated the function of GCNF using a mix of genetic and molecular biology methods. They compared transgenic mouse models producing a shortened version of GCNF without repressor activity to wild-type mice (Kavarthapu and Dufau, 2015). They also employed chromatin immunoprecipitation (ChIP) tests to identify GCNF genomic targets and study how they affect gene expression. The research showed that GCNF acts as a transcriptional regulator, suppressing the expression of Hox genes and pluripotency maintenance genes like Nanog and Oct4 (Hay et al., 2004). The researchers discovered that GCNF binds to regulatory areas of these target genes, attracting co-repressors and promoting chromatin remodeling, which results in gene expression suppression. Furthermore, the study indicated that GCNF plays an important role in maintaining the balance between pluripotent and differentiated cell states. For differentiation to begin, GCNF-mediated suppression of pluripotency genes is required (Ding et al., 2015). The researchers discovered that GCNF is downregulated throughout lineage determination, indicating that GCNF-mediated suppression of pluripotency genes is only required early in differentiation (Gu et al., 2005). Overall, the work clarified the mechanism of action of GCNF in the transcriptional control of genes involved in cellular differentiation and development. The research discovered that GCNF operates as a transcriptional repressor, controlling the balance of cells’ pluripotent and differentiated states. It has important implications for understanding embryonic development and finding possible targets for therapeutic approaches in disorders requiring abnormal gene expression regulation. This gene regulates cell growth and differentiation, and various extracellular signals, including growth factors and cellular stress, induce its expression. The leucine zipper transcription factor that regulates c-fos is called Fos, and it forms a heterodimer with another transcription factor called Jun to activate the c-fos promoter (Ahn et al., 1998). Several studies have investigated the role of leucine zipper transcription factors in gene regulation. For example, a study of the function of a leucine zipper transcription factor called GCN4 in yeast. The authors found that GCN4 regulates the gene expression in amino acid metabolism and that changes in its availability modulate its activity (Harding et al., 2000). Another study investigated the function of a leucine zipper transcription factor called AtbZIP11 in *Arabidopsis thaliana*. According to one study, abscisic acid (ABA), a plant hormone critical in response to diverse environmental stresses, induces AtbZIP11 (Yoshida et al., 2010). The scientists discovered that the expression of AtbZIP11 was increased in Arabidopsis plants treated with drought and high-salinity stress, confirming its significance in the abiotic stress response (Yoshida et al., 2014). Further, to study the role of AtbZIP11 in stress response, the researchers created transgenic Arabidopsis plants with both AtbZIP11 overexpression and AtbZIP11 under expression. The scientists discovered that AtbZIP11 modulates the expression of numerous genes involved in ABA production and signaling, osmotic stress response, and reactive oxygen species scavenging by studying the expression patterns of genes involved in abiotic stress response in both transgenic plants (Floris et al., 2009). Leucine zipper transcription factors (TFs) are useful in biotechnology they may be utilized to tailor cell behavior. Cancer cells’ proliferation can be slowed, for instance, by manipulating their gene expression levels. The researchers started by looking at ATF3 expression levels in breast cancer cell lines with different metastatic potentials. They discovered that highly metastatic breast cancer cells had much lower levels of ATF3 expression than less metastatic cells, indicating that ATF3 may play a role in limiting breast cancer cell metastasis. (Wang H. et al., 2021). To test this notion, the scientists used siRNA targeting ATF3 in lowly metastatic breast cancer cells to undertake knockdown tests. They discovered that ATF3 deficiency boosted cell motility, invasion, and metastasis both *in vitro* and *in vivo* (Xie et al., 2021). On the other hand, overexpression of ATF3 in highly metastatic breast cancer cells reduced metastasis *in vivo*, So these findings imply that ATF3 acts as a tumor suppressor in breast cancer by preventing cancer cell spread (Kwok et al., 2009). The researchers next looked at the molecular processes that underpin ATF3’s function in breast cancer metastasis. They discovered that ATF3 increases the expression of the MET tyrosine kinase, which is known to control cell motility and invasion. They also discovered that in breast cancer cell lines and clinical samples, MET expression was adversely linked with ATF3 expression (Shi et al., 2018). Furthermore, they demonstrated that MET overexpression could reverse the metastatic phenotype caused by ATF3 knockdown. Finally, the authors suggested that modulating ATF3 expression or its downstream targets, such as MET, might be a potential therapeutic method for breast cancer therapy, especially in patients with severe metastatic illness (El-Tanani et al., 2022). Overall, this study sheds light on the function of ATF3 in breast cancer metastasis and lays the groundwork for future research into the therapeutic targeting of ATF3 or its downstream targets for breast cancer therapy. Gene expression regulation is important for many animals and biological processes, and leucine zipper transcription factors are a big part of this. As more research is done, scientists will learn more about how these proteins work and how they could be used in biotechnology and medicine.

### 5.1 The role of leucine in epigenetics

Leucine is a crucial amino acid for protein synthesis, energy consumption, and cell communication. The mTOR pathway, which controls cell growth, proliferation, and survival, is one of the primary signaling routes in which Leucine participates (Nie et al., 2018). Leucine activates mTOR, leading to increased protein synthesis and cell growth. Recent studies have also implicated Leucine in epigenetic mechanisms contributing to growth and development (Jung et al., 2021). In the field of epigenetics, changes in gene expression are studied that do not entail alterations to the DNA sequence (Mohamad et al., 2019). Epigenetic alterations are heritable and may be altered by variables, including nutrition and lifestyle. Leucine has been demonstrated to influence epigenetic alterations, including histone modifications. Histones are proteins that aid in packaging DNA into chromatin, and changes to histones may affect how genes are expressed (Hasan, 2020). Leucine supplementation can improve muscle protein synthesis in older adults, and epigenetic modifications may mediate this effect (Koopman and van Loon, 2009). One study investigated the effects of leucine supplementation on the epigenetic regulation of growth hormone [GH] in mouse liver (Xie et al., 2022). Supplementation with leucine altered the expression of several genes involved in GH regulation in mouse liver, as shown by an upregulation of genes involved in GH synthesis and secretion and a downregulation of genes involved in GH breakdown (Le Roith et al., 2001). Researchers looked at the activation of many signaling pathways, including the mTOR signaling pathway, that is known to be involved in GH regulation to deduce the molecular mechanism behind these effects. Leucine is known to stimulate the mTOR pathway, a major regulator of cell growth and protein synthesis. The researchers discovered that leucine supplementation boosted the activation of the mTOR signaling pathway in mouse liver (Li et al., 2014). They specifically detected elevated phosphorylation of numerous important proteins involved in the system, including mTOR and its downstream targets, S6K1 and 4E-BP1. Notably, the researchers discovered that the observed alterations in histone H3 acetylation and GH gene expression were mediated by stimulation of the mTOR signaling system. A particular inhibitor of the mTOR system inhibited the effects of leucine supplementation on histone H3 acetylation and GH gene expression in mouse liver cells. The authors found that leucine supplementation increased the acetylation of histone H3, a modification associated with increased gene expression, at the GH promoter (Wang et al., 2012). They also observed an increase in the binding of the transcription factor cAMP response element-binding protein [CREB] to the GH promoter, which suggests that leucine supplementation may enhance GH expression through epigenetic mechanisms.

To find out how giving rats extra Leucine affects muscle growth and how epigenetics control the expression of myogenic genes. The scientists discovered that supplementing with leucine boosted the production of myogenic genes and the acetylation of histone H3 at their promoter regions (Sanchez and Sharma, 2009). So it is also observed an increase in the binding of the transcription factor myocyte (McGee and Hargreaves, 2004). So the study of acetylation found that Leucine can increase the acetylation of histones, leading to increased gene expression (Chen et al., 2004). In particular, Leucine has been shown to increase the acetylation of histones associated with genes involved in muscle growth and metabolism. In addition, Leucine has been shown to affect DNA methylation, another type of epigenetic modification (Amaral et al., 2014). One study found that leucine supplementation in obese mice led to changes in DNA methylation patterns in genes involved in energy metabolism (Liu and Sabatini, 2020). Overall, while the exact mechanisms by which Leucine affects epigenetic modifications are not fully understood, it is clear that Leucine plays a role in regulating gene expression through epigenetic mechanisms.

### 5.2 Leucine with seeing mechanisms

**FIGURE 3:**
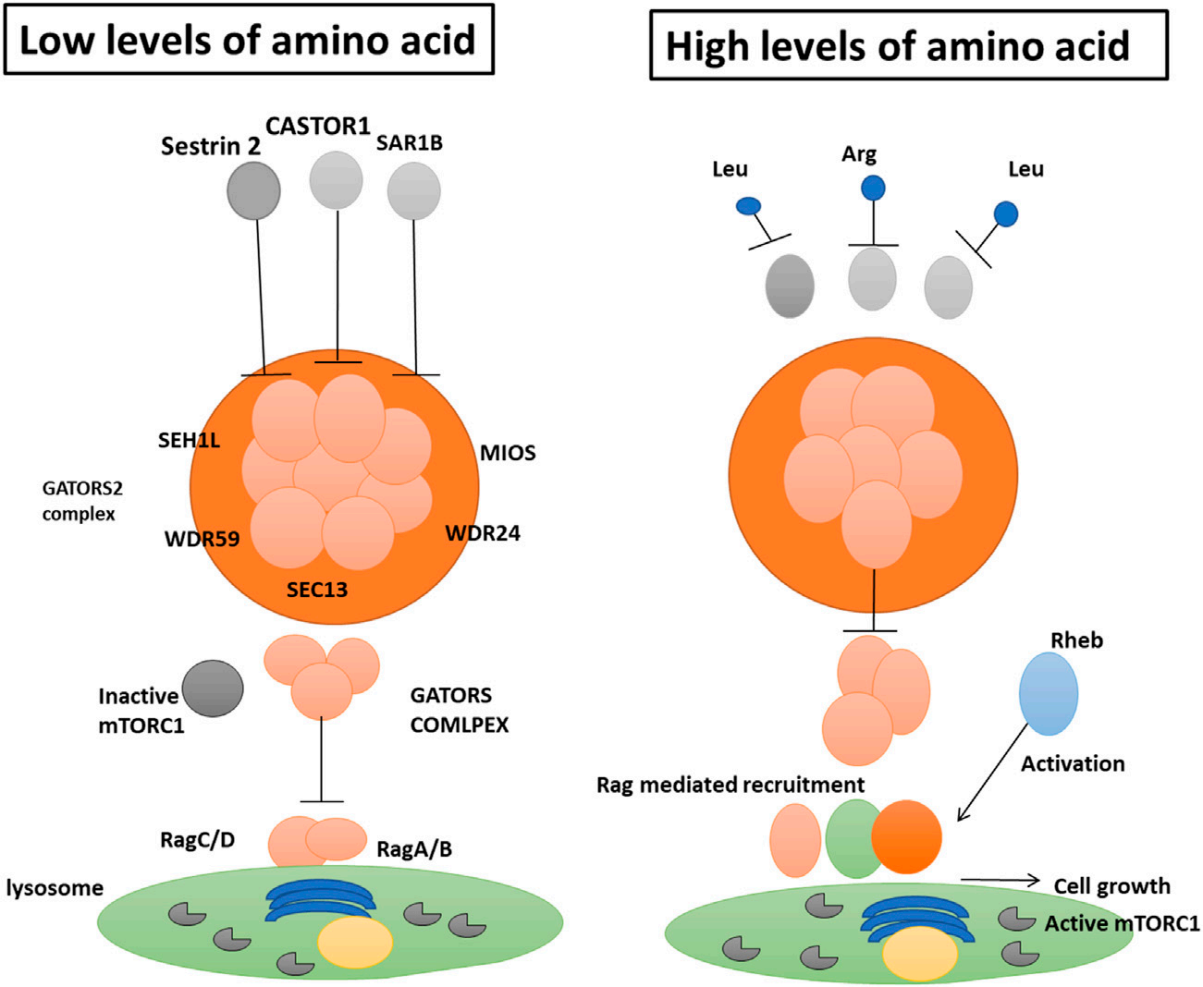
Intracellular amino acid availability regulates the activity of the mTORC1 complex, which controls cell growth. Chen et al. (Li et al., 2009) show that SAR1B protein plays a role in this process. When there are not enough amino acids, the protein complex GATOR2 is blocked by SAR1B, together with two other proteins, CASTOR1 and sestrin 2. (comprised of the amino acids SEC13, WDR24, WDR59, and MIOS). SAR1B inhibits MIOS, whereas sestrin 2 inhibits SEH1L. To prevent the inactive mTORC1 complex from being recruited to the lysosome, the complex GATOR1 (subunits not specified here) inhibits the enzymes RagA, RagB, RagC, and RagD.b, SAR1B and sestrin 2 are suppressed by an abundance of Leucine (Leu), while CASTOR1 is dampened by an abundance of Arginine (Arg). This allows GATOR2 to shut down GATOR1 effectively. After Rheb (a protein that senses components of cellular activity such as energy levels) unblocks the Rag enzymes, the Rag enzymes recruit mTORC1 to the lysosome. Under optimal circumstances, this pathway may integrate cellular data and set off growth.

Leucine is a powerful mTORC1 activator because it inhibits the function of the inhibitory protein sestrin 2 on the GATOR2 complex. This type of regulation connects cellular nutritional status and the amount of intracellular amino acids to cell growth control. The small GTPase enzyme SAR1B is another regulatory component in mammalian cells that detects Leucine (Liu and Sabatini, 2020). Researchers found that cells with low SAR1B expression were resistant to leucine deficiency and had their mTORC1 pathway activated. Like sestrin-2, the absence of Leucine triggers a physical interaction between SAR1B and GATOR2, which alters GATOR2’s function (Bröer and Gauthier-Coles, 2022). The authors demonstration that SAR1B interacts with an unalike GATOR2 component (the protein MIOS) than sestrin 2, which binds to the SEH1L protein. Once the intracellular concentration of leucine is high enough, SAR1B rearranges itself and separates from GATOR2. Following recruitment to the lysosome surface by the four Rag GTPase enzymes5 (RagA, RagB, RagC, and RagD), GTPase activates mTORC1 without GATOR2 (Figure 3) (Vellai, 2021). Therefore, GATOR1 relies on SAR1B to perform its inhibitory role on Rag GTPases. It permits mTORC1 to restrict to lysosomes even when leucine is scarce (Vellai, 2021). The scientists discovered that SAR1B and sestrin 2 recognize distinct facets of the structure of Leucine. While it was previously identified that sestrin 2 detects Leucine’s amino and carboxyl groups, they now disclose that SAR1B does so as well. The affinities of these two leucine receptors are distinct: The binding affinity of SAR1B is greater than that of sestrin 2.

Stress-responsive proteins SESTRIN1-3 control metabolic homeostasis in metazoans. (Bröer and Gauthier-Coles, 2022). Multiple mechanisms, including AMPK and the TSC complex activation, have been proposed to explain how sesterins inhibit mTORC1 (Taherkhani et al., 2021), (i) via inhibiting GDP dissociation from RAGA/B (a GDI [guanosine dissociation inhibitor] activity) and The cytoplasmic protein SESTRIN2 was shown to bind Leucine directly *in vitro* recently (Peng et al., 2014). Leucine does not activate mTORC1 in cells that produce a mutant of SESTRIN2 that cannot bind leucine. Leucine, along with isoleucine, methionine, and to a lesser extent, valine, prevents SESTRIN2 and GATOR2 from interacting in test tubes and living cells (González and Hall, 2017). [100] SESTRIN2 binds to and inhibits GATOR2 in cells that are deficient in Leucine (Chen et al., 2021). The generation of a GATOR2 binding-deficient mutant of SESTRIN2 in response to leucine deprivation demonstrates that SESTRIN2 controls mTORC1 through GATOR2 (González and Hall, 2017). SESTRIN2 dissociates from GATOR2 in response to leucine binding, resulting in mTORC1 translocation to the lysosome (ii) by binding and inhibiting GATOR2 from preventing mTORC1 lysosomal localization in response to amino acids (Liu and Sabatini, 2020). However, SESTRINS may inhibit mTORC1 in cells growing in media containing Leucine; hence their function as leucine sensors have been called into doubt (Eliopoulos et al., 2016). Researchers found that leucine administration enhanced sestrin2 expression in cultured muscle cells and mice, activating AMP-activated protein kinase [AMPK] and inhibiting mTOR signaling. In muscle cells and mice, inhibiting mTOR signaling was related to higher glucose absorption and better insulin sensitivity. (Lee et al., 2016). Another study reported that sestrin2 plays an essential role in regulating the favorable effects of Leucine on glucose metabolism in mice fed a high-fat diet (Kamei et al., 2020). The study involved feeding mice a high-fat diet supplemented with leucine or a control diet without leucine for 10 weeks. The researchers then examined the influence of leucine supplementation on glucose metabolism, insulin sensitivity, and gene expression relevant to metabolism in skeletal muscle tissue. The study’s findings indicated that leucine supplementation increased glucose tolerance and insulin sensitivity in mice fed a high-fat diet. Reduced blood glucose and insulin levels during glucose tolerance tests served as evidence of this, indicating that leucine may increase glucose metabolism even when consumed in conjunction with a high-fat diet. Furthermore, the researchers observed that leucine supplementation boosted the expression of multiple genes associated with glucose metabolism in skeletal muscle tissue, confirming the beneficial metabolic benefits of leucine. To evaluate the role of sesn2 in controlling the positive metabolic effects of leucine on glucose metabolism, the researchers analyzed the expression of sesn2 and discovered that leucine supplementation increased sesn2 expression in skeletal muscle tissue. This motivated the researchers to study the involvement of sesn2 in leucine control of glucose metabolism (Cangelosi et al., 2022). Supplementing these animals with Leucine increased sestrin2 expression and activity, which turned on AMPK and made skeletal muscle better at absorbing glucose and responding to insulin (Bifari and Nisoli, 2017). The mechanism by which sestrin2 suppresses mTOR signaling is unknown; however, it is assumed to entail AMPK activation and Rag GTPase inhibition, which are required to activate mTORC1 by amino acids such as Leucine (Rabanal-Ruiz et al., 2017). Leucine deprivation causes Leucine to dissociate from SESTRIN2, although it is unclear if other variables control this process. So sestrin2 is a protein that plays an essential role in mediating the beneficial effects of Leucine on glucose metabolism and insulin sensitivity. Leucine stimulates sestrin2 expression and activity, inhibiting mTOR signaling and improving glucose uptake and metabolism. These findings suggest that sestrin2 may be a potential therapeutic target for preventing and treating metabolic disorders such as type 2 diabetes.

## 6 Immunity

Multiple studies conducted in the 1970s showed that immune function was impaired in those who did not consume enough Leucine or other BCAAs. (Cynober and Harris, 2006). Leucine is an essential amino acid that plays a crucial role in many physiological processes in the human body, including protein synthesis, muscle growth, and immune function. In particular, Leucine has been revealed to play a critical role in initiating and regulating immune responses (Marc Rhoads and Wu, 2009) and activates the mammalian target of the rapamycin [mTOR] signaling pathway, which regulates cell growth, metabolism, and immune function. mTOR signaling is required for immune cell development and function, including T, B, and natural killer [NK] cells (O’Brien and Finlay, 2019). The supplementation of Leucine has been shown to increase the production of perforin and granzyme B, two key molecules that NK cells use to destroy infected or cancerous cells (Bratke et al., 2005). Leucine also plays an essential role in controlling the synthesis and operation of antibodies, B-cell-produced proteins crucial for defending the body against infections. Leucine has been demonstrated to increase the production of immunoglobulin A [IgA], an antibody essential for defending the body’s mucous membranes (Terezhalmy et al., 1996). Human peripheral blood mononuclear cells [PBMCs] and sheep splenocytes [Spleen cells] were used to examine the immunomodulatory effects of leucine metabolites [HMB, KIC] (Cruzat et al., 2018). Studies have shown that leucine supplementation can enhance immune function in various ways. For example, it can increase the production of T cells, important immune cells that help fight infections. The effects of leucine supplementation on immunological function in mice should be investigated. They were particularly interested in the effect of leucine on T-cell generation and activation, both of which are important components of the immune system. The researchers fed mice a leucine-supplemented meal while a control group was offered a conventional diet without supplementation. T-cell abundance and activation in the mice were then examined. The researchers discovered that giving mice leucine enhanced the number of T cells, namely, CD8^+^ and CD4^+^ T cells (Song et al., 2020). These T cells were then evaluated to see if they could develop into effector cells, which are crucial in combating infections (Song et al., 2020). According to the findings, leucine supplementation increased T-cell development into effector cells, indicating an improved ability to protect against infections. Furthermore, the researchers discovered that leucine supplementation increased T-cell proliferation, allowing for a faster and more effective response to infections (Bermon et al., 2017). According to the study, Leucine was also shown to enhance the production of cytokines, which are essential signaling molecules that govern immunological responses. To clarify, the researchers discovered that leucine supplementation improved T-cell proliferation and differentiation and their ability to create cytokines, which are essential signaling molecules that govern immune responses (Yoon et al., 2018). The researchers discovered that leucine supplementation increased the synthesis of interferon-gamma (IFN-), interleukin-2 (IL-2), and tumor necrosis factor-alpha (TNF-), all of which are cytokines linked with improved immune function and infection protection (Li et al., 2018). Overall, the findings imply that supplementing with leucine can improve immune function in mice by enhancing T-cell generation, differentiation, and activation. The study found that the mechanism of leucine-induced immunological enhancement includes the activation of cellular signaling pathways that control T-cell proliferation and differentiation. Furthermore, leucine has been shown to have anti-inflammatory properties, which can help to reduce inflammation in the body and support immune function. Inflammation is a natural response to infection and injury, but chronic inflammation can impair immune function and increase the risk of disease (Eddleston et al., 2007). Leucine plays a critical role in maintaining immune function, and its supplementation may have therapeutic potential for improving immune health. However, it is essential to consult a healthcare professional before starting any new supplements or dietary changes.

## 7 Conclusion

**FIGURE 4:**
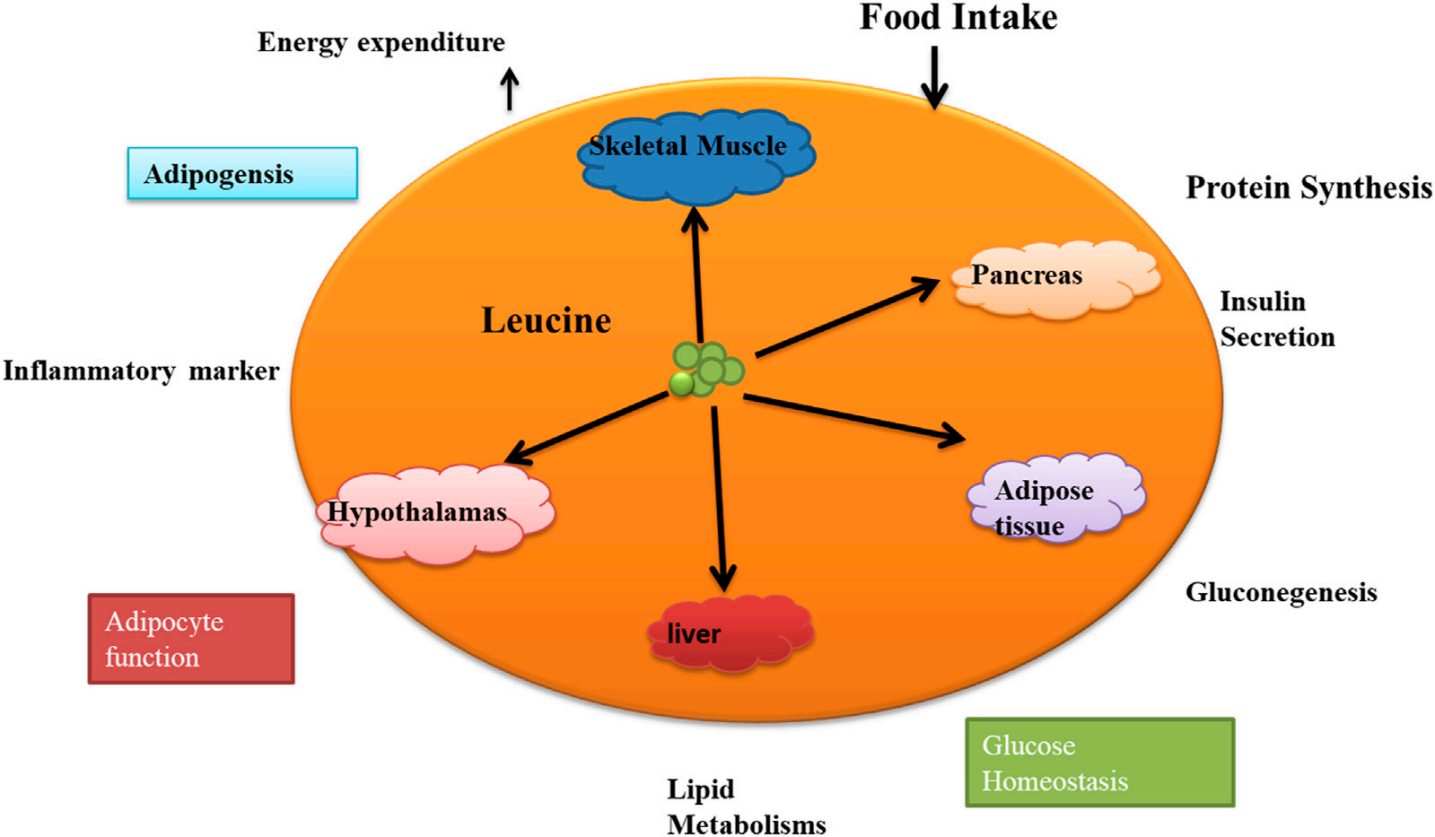
The regulation of glucose homeostasis and energy balance may be affected by leucine supplementation. This strategy summarizes the information presently known on the possible effects of leucine supplementation in different tissues.

Leucine has been studied extensively because of its function as a nutritional signal activating the mTORC1 pathway in various functions, including muscle function, insulin resistance, and immune response activation. In addition to being critical for muscular growth, getting enough leucine is important for your health and wellbeing in general. It aids in the upkeep of strong bones, controls blood sugar, and promotes a robust immune system. Inadequate leucine intake has been linked to various health issues that may reduce quality of life. To maintain a healthy and robust body, consuming sufficient amounts of leucine-rich meals or supplements is essential. Putting effort into your health now will pay you in the long run. Leucine is essential for nutrition and physiological activities such as metabolism of carbohydrates and fats, protein synthesis, digestive tract wellness, and immune system performance. The potential effect of leucine is brief below (Figure 4).

The potential therapeutic effects of leucine supplementation have been examined in a various scenario, as a dietary supplement for the treatment of obesity and type 2 diabetes, due to leucine’s ability to affect a variety of physiological processes through mTOR and perhaps other signaling pathways. Leucine is an essential amino acid that regulates protein synthesis and cellular metabolism. Lysosomes can detect high leucine levels and generate an anabolic response, increasing protein synthesis and cell development. However, when leucine levels are low, lysosomal activity may be inhibited, favoring catabolism and energy conservation. Although the precise mechanisms governing lysosomal leucine sensing remain unknown, new investigations have identified many signaling pathways and molecular sensors, including mTOR and the Rag GTPases. Overall, the involvement of lysosomes in leucine sensing shows the complicated interplay between nutrition availability and cellular metabolism, which could have significant implications for developing innovative therapeutics for metabolic disorders and aging-related diseases.

The possibility that leucine is one of the “active components” in high-protein diets has prompted interest in studying its potential medicinal uses. Many studies have shown that eating a diet rich in protein may help reduce weight and regulate blood sugar. Research has shown that increasing dietary leucine intake can improve glucose tolerance, reduce insulin resistance, and promote weight loss, particularly in individuals with obesity or type 2 diabetes. These effects appear to be due to leucine’s ability to stimulate thermogenesis, increase fatty acid oxidation, and improve insulin signaling, which can promote healthy glucose metabolism and weight loss. Based on the data presented, leucine supplements are not expected to be useful in treating obesity. Finally, the therapeutic potential of leucine for enhancing glucose homeostasis was investigated. However, the mechanisms by which leucine supplementation improves glucose tolerance remain unclear and may be somewhat dependent on weight loss. It is worth noting that studying the molecular mechanisms of leucine action in whole animals (*in vivo*) proves challenging due to its complexity. Consequently, researchers use cell culture methods (*in vitro*) as a valuable approach to unravel the intricate mechanisms underlying leucine action. Nevertheless, it is important to acknowledge certain limitations associated with the use of cell culture systems. *In vitro* systems rely on immortal cell lines that differ from normal cells, which necessitates the validation of *in vitro* findings through *in vivo* methods. Future prospective studies on the role and mechanism of leucine in regulating animal growth and development through mTOR have the potential to make significant contributions to animal health, welfare, and production, as well as to our understanding of the fundamental mechanisms underlying muscle growth and tissue repair in animals.

## Figures and Tables

**Table:** 

**4E-BP1**	Eukaryotic translation initiation factor 4E-binding protein 1
**AA**	Amino acid
**ACC**	Acetyl-CoA carboxylase
**ACOX1**	Acyl-CoA oxidase 1, palmitoyl
**Akt/PKB**	Protein kinase B
**AMPK 5′**	Adenosine monophosphate-activated protein kinase
**AS160**	Akt substrate of 160 kDa
**ATF4**	Activating transcription factor 4
**CAMKII**	Calcium/calmodulin-dependent protein kinase II
**CKD**	Chronic kidney disease
**COX**	Cytochrome C oxidase subunit
**CPT-1B**	Carnitine palmitoyltransferase 1
**CrAT**	Carnitine O-acetyltransferase
**CRP**	C- reactive protein
**CytC**	Cytochrome C
**EDL**	Extensor digitorum longus
**EE**	Energy expenditure
**eEF**	Eukaryotic translation elongation factor
**eIF**	Eukaryotic translation initiation factor
**ER**	Extracellular signal-regulated kinase
**FAS**	Fatty acid synthase
**FFA**	Free fatty acid
**FOXO1/3a**	Forkhead box O1/O3a
**GβL**	G protein beta subunit-like
**GCN2**	General control nonderepressible 2
**GHR**	Growth hormone receptor
**GLUT4**	Glucose transporter 4
**GS**	Glycogen synthase
**GSH/GSSG**	Glutathione
**GSK3**	Glycogen synthase kinase 3
**HbA1c**	Glycated hemoglobin
**HOMAIR**	Homeostatic model assessment of insulin resistance
**Hspd1**	Heat shock 60 kDa protein 1
**hVps34**	Human vacuolar protein sorting 34
**IGF-1**	Insulin-like growth factor
**1 IGFBP**	Insulin-like growth factor-binding protein
**IL-1β/6/15**	Interleukin-1β/6/15
**IR**	Insulin receptor
**IRS1**	Insulin receptor substrate 1
**LAT1**	Large neutral amino acid transporter
**LC3I/II**	Microtubule-associated protein 1A/1B-light chain 3 I/II
**MAFbx**	Muscle atrophy F-box
**MAPK**	Mitogen-activated protein kinase
**MCAD**	Medium-chain acyl-CoA dehydrogenase
**MCP1**	Monocyte chemotactic protein 1
**MDA**	Malondialdehyde
**MHCII**	Major histocompatibility complex class II
**Mnk1**	MAP kinase-interacting serine/threonineprotein kinase 1
**MnSOD**	Mitochondrial antioxidant manganese superoxide dismutase
**mTOR**	Mammalian target of rapamycin
**MuRF1**	Muscle RING finger 1
**NAMPT**	Nicotinamide phosphoribosyltransferase
**NO**	Nitric oxide
**NRF**	Nuclear respiratory factor
**OCR**	Oxygen consumption rate
**PCr**	Creatine phosphate
**PDK**	Phosphoinositide-dependent kinase
**PGC-1α**	PPAR-gamma coactivator-1 alpha
**PI3K**	Phosphatidylinositol-4,5-bisphosphate 3-kinase
**PIP2**	Phosphatidylinositol-4,5-bisphosphate
**PIP3**	Phosphatidylinositol-3,4,5-trisphosphate
**PP2A**	Protein phosphatase 2
**PPAR**	Peroxisome proliferator-activator receptor
**PSMB7**	Proteasome subunit beta type-7
**PRAS40**	Proline-rich Akt substrate of 4 kDa
**REDD2**	Regulated in development and DNA damage responses 2
**RER**	Respiratory exchange ratio Rheb Ras-homolog enriched in the brain
**RG**	Red gastrocnemius
**RQ**	Respiratory quotient
**S6**	Ribosomal protein 6
**S6K**	Ribosomal S6 kinase
